# Development and Use of Health-Related Technologies in Indigenous Communities: Critical Review

**DOI:** 10.2196/jmir.7520

**Published:** 2017-07-20

**Authors:** Louise Jones, Kristen Jacklin, Megan E O'Connell

**Affiliations:** ^1^ Centre for Rural and Northern Health Research Laurentian University Sudbury, ON Canada; ^2^ Medical Anthropology Human Sciences Division Northern Ontario School of Medicine Sudbury, ON Canada; ^3^ Rural and Remote Memory Clinic Department of Psychology University of Saskatchewan Saskatoon, SK Canada

**Keywords:** Indians, North American, Canada, telemedicine, self-help devices, needs assessment, aging

## Abstract

**Background:**

Older Indigenous adults encounter multiple challenges as their age intersects with health inequities. Research suggests that a majority of older Indigenous adults prefer to age in place, and they will need culturally safe assistive technologies to do so.

**Objective:**

The aim of this critical review was to examine literature concerning use, adaptation, and development of assistive technologies for health purposes by Indigenous peoples.

**Methods:**

Working within Indigenous research methodologies and from a decolonizing approach, searches of peer-reviewed academic and gray literature dated to February 2016 were conducted using keywords related to assistive technology and Indigenous peoples. Sources were reviewed and coded thematically.

**Results:**

Of the 34 sources captured, only 2 concerned technology specifically for older Indigenous adults. Studies detailing technology with Indigenous populations of all ages originated primarily from Canada (n=12), Australia (n=10), and the United States (n=9) and were coded to four themes: meaningful user involvement and community-based processes in development, the digital divide, Indigenous innovation in technology, and health technology needs as holistic and interdependent.

**Conclusions:**

A key finding is the necessity of meaningful user involvement in technology development, especially in communities struggling with the digital divide. In spite of, or perhaps because of this divide, Indigenous communities are enthusiastically adapting mobile technologies to suit their needs in creative, culturally specific ways. This enthusiasm and creativity, coupled with the extensive experience many Indigenous communities have with telehealth technologies, presents opportunity for meaningful, culturally safe development processes.

## Introduction

The Canadian population is aging rapidly. In July 2015, Statistics Canada reported that people aged above 65 years outnumbered children below the age of 14 years. The trend of an aging Canada is projected to continue: by the year 2024, over 20% of the total population will be over the age of 65 years [1]. National bodies such as the Canadian Medical Association have expressed concern about increasing demands on Canadian health care systems due to the aging of the population, which will result in an increase in age-related disorders such as dementia [2]. Within the Canadian population, Indigenous populations now exceed one million and are growing at a rate 6 times greater than that of the population as a whole [3]. The number of Indigenous adults aged 60 years and above is projected to increase 3.4 times from 2006 to 2031, resulting in over 184,000 older Indigenous adults [4]. Aging within Indigenous communities interacts with social inequities; consequently, older Indigenous adults may be more likely to require regular and specialized health care. For example, First Nations communities in Canada have higher rates of chronic conditions such as diabetes and heart disease [5], impacting quality of life and need for health services. These same communities often have insufficient financial resources to address growing health concerns and may be geographically disadvantaged in terms of access to facilities and providers [6]. This geographic concern is compounded by findings that the majority of older Indigenous adults prefer to grow older in their own homes, known as “aging in place” [7]. Aging in place is also more cost effective than long term care for both families and governments [8] and aligns with recent findings suggesting that family caregiving models in Indigenous communities are preferred. Indigenous caregiving models are not only more robust than those of the general population but are culturally grounded and present unique health and social service needs [9].

Literature concerning technological innovations for aging in place has developed significantly over the past several years, and meaningful user involvement has been identified as critical to adoption [10-12]. Comparable Indigenous-specific literature is scarce, despite the fact that the development of novel assistive technologies has potential to support health care delivery and aging in place in Indigenous communities [13]. User needs exploration is important to promote any end-use adoption of technology, but it is particularly important for Indigenous populations where user needs may differ from those espoused by older adults from the majority culture or other marginalized cultures, and all information technology is culturally bound [14]. Development of culturally safe, useful technology can only be developed by collaborative participation with Indigenous end users [15]. Consequently, the purpose of the paper was to explore the current state of the available literature concerning use, adaptation, and development of assistive technologies for health purposes by Indigenous individuals and communities.

## Methods

### Methodology

Initially, the research team endeavored to systematically review available literature concerning older Indigenous adults and their use of technology for health purposes. Informal searches presented keyword possibilities such as “assistive technology” and indicated that there may not be sufficient literature specific to older Indigenous adults available for systematic review. Age parameters within our chosen population were expanded to accommodate this concern, and the research question was finalized within a critical review framework for the purposes of best highlighting gaps in the literature. Working within Indigenous research methodologies also affected the search method. Research centering Indigenous voices and experiences was prioritized, and Indigenous-centered, nonacademic literature was evaluated and accepted as equal to academic sources. This practice is drawn from Indigenous research scholarship and is sometimes referred to as “choosing the margins” [16]. Indigenous cultures also uniquely experience colonization, a lived reality distinct from racism or discrimination experienced by other groups; consequently, articles about adaptation with other marginalized groups were not included. Due to the paucity of published literature meeting the search criteria, sources were sought on multiple platforms and practices such as hand searching were employed to ensure that all relevant studies were captured.

### Search Strategy

Searches of peer-reviewed, academic literature and gray literature on assistive technologies and Indigenous people and communities were completed in February 2016. In the academic literature search, keywords related to Indigenous (“Aboriginal,” “Indigenous,” “Inuit,” “Métis,” “First Nation,” “Native American,” and “American Indian”) and assistive technologies (“assistive technology,” “assistive devices,” “ehealth,” and “mHealth”) were combined and the search was conducted on several databases: MEDLINE, PubMed, PsycInfo, AMED, EMBASE, CINAHL, Social Work Abstracts, Social Services Abstracts, ProQuest Health, and the Cochrane Database of Systematic Reviews. Based on findings from this search, hand searching was also completed in both references of found articles and in the following journals: Telemedicine and Ehealth, Journal of Circumpolar Health, Rural and Remote Health, Journal of Assistive Technologies, and Journal of Social Work in Disability & Rehabilitation *.* Due to an overabundance of articles regarding telehealth use and development in Indigenous communities, articles captured in the search focused on telehealth were limited to Canadian and post-1990. To capture the diversity of available technologies beyond telehealth, international sources are included, primarily from the United States, New Zealand, and Australia; countries with similar colonial histories to Canada.

Gray literature was searched using the method from “Grey Matters: A Practical Search Tool for Evidence-Based Medicine” produced by the Canadian Agency for Drugs and Technologies in Health. This method was used to search the websites of the following parties: Alberta College of Family Physicians, Alberta Health and Wellness, Canadian Agency for Drugs and Technologies in Health, McGill University Health Centre, Newfoundland and Labrador Centre for Applied Health Research, Therapeutics Initiative, Australian Government Department of Health and Ageing, Australian Government Department of Health and Ageing Medical Services Advisory Committee, Monash Health Centre for Clinical Effectiveness, Institute of Technology Assessment, Agency for Healthcare Research and Quality, California Technology Assessment Forum, Centers for Medicare and Medicaid Services, Institute for Clinical and Economic Review, Alberta Medical Association, British Columbia Ministry of Health Services, Canadian Medical Association, University of Ottawa School of Rehabilitation Science, Academy of Medicine of Malaysia, Haute autorité de santé, and Alzheimer Society of Canada.

From these searches, 107 articles appeared to meet the search criteria and were selected for more detailed review. Upon detailed examination, 73 were excluded as they did not meet the search criteria, presenting no insight into Indigenous-specific use, adaptation, or development of technologies for health purposes. A total of 34 sources were indexed and summarized using bibliographic management software and content was coded to emergent themes.

## Results

### Data Analysis

There were few published studies on assistive technology use, development, or adaptation in Indigenous populations, globally. Disease-specific studies were limited (n=9), as were studies concerning older Indigenous adults (n=2). Results originated primarily in Canada (n=12), Australia (n=10), and the United States (n=9). Some studies originating in the United States found that assistive technology use in Indigenous communities was high compared with the wider American population [17,18], providing needed insight into experiences of use by this population. Studies also addressed different products: telehealth (n=8), mobile health [mHealth] (n=12), Web-based interventions (n=3), and assistive devices (n=7). The remaining 4 articles addressed considerations for development generally. Results were presented thematically: meaningful user involvement and community-based processes in development, the digital divide, Indigenous innovation in technology, and health technology needs as holistic and interdependent.

### Meaningful User Involvement and Community-Based Processes in Development

Several studies detailed the importance of developing technology with, as opposed to for, Indigenous communities. The purpose of developing with is not only to ensure that technology is relevant and useful but also to decolonize the development process [14,15,18-21]. This means involving users, caregivers, health professionals, and elders or original knowledge keepers (as appropriate) at the conception of the design and throughout all development and testing phases. Within studies considering development of technology, some researchers presented models or recommendations for meaningful engagement around technology with Indigenous communities, whereas others discussed challenges.

Maar and colleagues (2010) discussed the development of eHealth (also known as telehealth) with Indigenous communities in Canada. In their study, a participatory action research (PAR) approach was blended with Indigenous research methods to identify priority areas for Indigenous health research. The research team emphasized the often negative impacts that research and expert positioning have had on Indigenous communities, reminding readers that these impacts have contributed significantly to the historic and ongoing colonization of Indigenous peoples. They suggested the engagement of individuals with real-world understanding and meaningful, lived connections to Indigenous communities rather than those coming from outside the community. Also emphasized was the importance of communities, not researchers, developing research priorities and that researchers should take the time needed to hear all voices to achieve consensus. Their conceptual model places community advisory councils at the center of the research process [15].

Community advisory councils were also at the center of a study by Davies and colleagues (2015) on the development of a culturally appropriate mobile phone app for the prevention and management of hepatitis B for Indigenous Australians. The app was piloted in a community of just over 2100 people with overcrowded homes and limited amenities. Researchers used storyboarding to present ideas and seek feedback from an advisory council after initial interviews. They took the time to ensure community needs were accurately reflected, with some storyboards undergoing more than 20 versions before approval. The app was then developed and translated into the local Indigenous language, which required a significant investment in time involving back translation and testing with fluent community members. When researchers felt the app was finally ready for market, prototypes were launched in the community and feedback was sought. This process was repeated 4 times before the app was correctly tailored to community needs [14]. Another study for the adaptation of a model of remote monitoring for use by American Indian veterans with post-traumatic stress disorder described a similar engagement process and suggests employing a cyclical model in which user feedback continually informs cultural adaptation, rather than a linear process [20].

Similarly, Ratliffe and colleagues (2012) reported on a series of case studies conducted in the Pacific Islands and presented best practices and barriers for adaptation. Best practices included establishment of support networks for users, employing creativity when adapting the home environment, resource sharing when possible, and accepting an iterative change process [22]. Furthermore, multiple studies recommended meaningful involvement of the community in the development stages for successful adoption [15,21,23].

### The Digital Divide

Despite the best practices presented by these participatory approaches, significant challenges remain present for communities and for those developing “with.” Often identified was the “digital divide,” wherein some individuals and communities have greater access to the Internet, broadband, and cell towers than others. This is a continuing problem in Indigenous communities in Canada [15]. Morey (2007) suggests an expansion of this definition to include pronounced lack of Web-based content specific to the cultures and languages of marginalized communities and promotes the concept of “cultural usability” as a development concept to counteract this divide. Questions to consider under the cultural usability umbrella include whether content is relevant to the community, if the illnesses featured are concerns to the users, and whether or not suggestions for prevention and management are realistic given geographic location and socioeconomic status [24]. This definition can be further expanded to include affordability of technologies for individuals and community, a barrier that was cited in multiple studies [13,18,22].

A key aspect to the digital divide is remoteness or rurality, a concept explored in greater detail in 3 studies [13,15,19]. Arnold (2009) demonstrates this reality for remote and rural Aboriginal and Torres Strait Islander communities in Australia. She suggests considerations for development and implementation include being aware of distance to nearest maintenance personnel and part stores, as this will determine cost and convenience for the user. Arnold also notes that environmental factors that impact the use and implementation of the technology need to be considered. For example, the roads may not be paved, the user may spend time in the bush, and coastal areas will accelerate rusting of technology and communication infrastructure. Furthermore, older community members often live with extended family, and as such, the technology needs to adapt to not only the user’s need, but also to any family members who are sharing the same living spaces. Further data on the importance of adaptation was detailed by Reisinger and Ripat (2014) who conducted a series of talking circles with US-based Navajo users and assistive device providers. Best practices identified by users included adapting assistive devices to their environment, especially with consideration to frequent outdoor use and overcrowded housing. This same community also expressed concerns regarding insufficient infrastructure to support new technologies [18]. Recommendations were varied, but a top suggestion was to employ lengthy and rigorous trial periods for new devices [18]. Primarily, working with the user during as many stages of implementation as possible is advised. Maar and colleagues (2010) reemphasize the complexity and diversity of Indigenous communities and the importance of a good engagement model as vital to managing adaptation and adoption of technology challenges.

Telehealth has been recommended for years as a technological solution to mitigate Indigenous health care disparities: health care providers do not always live where service users do, particularly if users live on reserve or in remote locations [21,25]. Nevertheless, research in telehealth use with Indigenous populations produces mixed results. One study found telehealth and remote monitoring to be as effective as conventional care [26], whereas another cautioned that telehealth should only ever be used as a complement to conventional care and that some service users should not even be considered for remote services due to the severity of their conditions [27]. Furthermore, although many participants were pleased with increased access to health care [23,28], one Ontario, Canada study found that almost a third of patient participants felt negatively about telehealth services as a whole, with most patients describing concerns about the cultural appropriateness and the privacy of the service [28], indicating poor cultural usability. Another challenge presented in the literature is a lack of training for health care providers using telehealth; Gibson and colleagues (2011) found that only 16% of providers had received any training on the technology they were using. This may contribute to a finding by Sidhu (2012) in which 45 cancer care professionals in British Columbia, Canada, were surveyed regarding perceptions of telehealth; most felt it less beneficial than conventional care. Finally, the digital divide presented as a physical challenge in some Ontario communities where broadband networks were actually insufficient to support the technology [15,23,28]. Despite these challenges, use remains fairly widespread. Factors found to contribute to more successful implementation and use of telehealth services were identified in 2 studies. Mah (2011) found that patients were more likely to engage in telehealth for the management of a specific illness than for general health care. Also, if technology was perceived as easy to use, successful uptake was also more likely [29].

Unlike telehealth, mHealth is a relatively new platform for the delivery of health care interventions. The term “mHealth” is used to describe mobile phone technologies used for health purposes. However, recommendations for enhanced cultural usability remain relevant. Emerging rural and Indigenous research suggests that the landscape of the digital divide is changing rapidly. Whereas Morey (2007) cites the digital divide as a challenge facing communities, other researchers view it as an opportunity to study innovation. In Canada, Maar and colleagues (2016) are completing the DREAM-GLOBAL project, in which they are developing culturally safe text messages (short message service, SMS) for hypertension management in Indigenous populations. A major finding was that messages should adapt to local socioeconomic and geographic conditions. They also concluded that cultural safety is essential to success [30]. Similarly, 2 Australian studies provided recommendations to occupational therapists working with Aboriginal and Torres Strait Islander people, such as accessing Indigenous health workers and seeking out cross-cultural training [19,31].

### Indigenous Innovation in Technology

Brusse and colleagues (2014) describe the enthusiastic uptake of mobile technology and social media among Indigenous peoples in Australia, stating that “In the past 5 years, affordable mobile phones with camera and messaging functions have spawned a ‘mobile phone culture’ in some remote areas, where messages, pictures, and video clips flow freely among and between communities, often in culturally unique and creative ways” [31]. Surveys of health care consumers in New Zealand indicate similar enthusiasm in Maori communities. Interest in mHealth interventions for weight loss was higher among rural Maori and young people compared with the general population [32]. Interest in a text messaging-based intervention for alcohol abuse was also viewed favorably by Maori participants, provided there were considerations for cultural relevance [33]. Culturally safe text-based interventions for retention in a clinical trial were found to be effective with Maori participants by other researchers [34]. Similar results have been found in Indigenous communities near La Paz, Bolivia; most participants texted regularly and were open to mHealth interventions, provided Indigenous language preferences were taken into account [35]. Indigenous language use was mentioned as a key factor for successful distribution and uptake in 3 other studies [14,36,37]. Other factors for success include the use of real people and stories to market and guide the user through the product, the use of more visuals than text, considerations for gender differences, and making the product free and available on multiple platforms [14,38].

### Health Technology Needs as Holistic and Interdependent

A major finding of the review is that Indigenous users of technology for health were not concerned with enhancing independence but rather interdependence. This includes recognizing that Indigenous perceptions of health may differ greatly from Western perceptions. In some instances, community-based, decision-making processes may be preferred to individualized systems [13,19], and independence may not be the ultimate goal of the user [19,39]. Users readily adopted technologies that included family and community in their health care, as well as technologies that fostered closer relationships with health care providers. For example, researchers in Nigeria have piloted a tool that does real-time machine translation between patients speaking Yoruba, an Indigenous language, and English-speaking doctors during remote consultations. Patients also have the option of selecting symptoms from a designated list, which prompts memory. Both medical personnel and patients demonstrated high levels of satisfaction with the service [37]. Recently, researchers in Australia evaluated the new AIMhi Stay Strong app, which is a mental health evaluation tool for health care providers to use on home visits with Aboriginal and Torres Strait Islander patients. Providers interviewed felt the app encouraged them to have more in-depth conversations with patients. Some noted that it helped even out the power relationship between provider and patient, as the patient could see what the provider was doing on the app and felt involved in the assessment process [36]. In Nain, Newfoundland, researchers studied a remotely controlled robot that could assess and provide some treatment. The robot was always with a nurse and an interpreter. Futhermore, 95% of patients indicated that they would use the robot again. Researchers recommended the robot be used full time, managed by nurses on site [40].

Another example of this relationship-based view of health and technology was found in the 3 studies regarding Web-based interventions for the management of diabetes by Native American users. All 3 studies found increases in self-management behavior [41-43], whereas 2 found improvements in blood glucose levels and other physical measures of health status as a result of the intervention [42,43]. Robinson and colleagues (2011) also determined that the more frequently and personally the users interacted with their health care providers via the website, the more carefully they monitored and managed their blood glucose levels. Jernigan and Lorig (2011) included a qualitative component to their study, and participant feedback indicated that the most valued aspect of the program by users was the culturally specific peer support. Many users described a feeling of safety derived from being among other Native American participants, pointing out that they did not need to watch their language or explain concepts specific to the Native American experience.

Furthermore, many Indigenous communities viewed health as more than simply physical, and this was reflected in their diversification of existing technologies. Recommendations for successful adoption of telehealth include the inclusion of traditional practices and beliefs in telehealth care [13,23]. Molyneaux and O’Donnell (2009) further suggest that the use of the technology be diversified and cite communities that are using the technology for a variety of purposes such as connecting loved ones for hospital visits, providing elders with social visits, staff training, health literacy education, programs for youth, and language sharing initiatives. One community discussed in their study had even invented the term “telespirituality” to describe consultations related to traditional medicine or ceremonial practices. They argue that this diversification demonstrates the value of the product to communities, which makes successful uptake more likely and involves multiple parties, establishing long term sustainability. The need for interdependence and holism was reflected in another American study where users wanted to see more frequent visits from community health representatives, peer support groups, and programs for assistive devices designed by and for users. Many mentioned the need for services to be provided in the Navajo language and with an understanding of traditional practices and ceremony [18].

## Discussion

### Principal Findings

Throughout the review, a number of pressing recommendations and major gaps were identified in the literature, providing insight into future research activities, policy change needed to address barriers, and positive innovation by Indigenous communities. Within each theme, the research team developed specific recommendations and identified concerns and gaps in knowledge. A pressing recommendation within the development theme emphasizes the importance of meaningful user involvement. Of note is that in spite of, or perhaps because of the digital divide, Indigenous communities are enthusiastically developing and adapting mobile technologies and social media to suit their needs, often in creative, culturally specific ways. This enthusiasm and creativity, coupled with the extensive experience many Indigenous communities have with technologies such as telehealth, presents an opportunity for meaningful, culturally safe development processes, as evidenced in recent work by Maar and colleagues [30]. Future research in this area should support these strengths and seek opportunities for culturally safe development, advocacy, and policy change.

Outside of Canada, mHealth apps appear to be emerging as a well-adopted technology in Indigenous communities, particularly within Australia and New Zealand. Canadian research in this area is significantly underdeveloped, perhaps also due to the above mentioned digital divide. Further study of perceptions of mHealth in Canadian Indigenous communities is needed. Web-based interventions were similarly not visible in the Canadian literature but presented good results in the few American studies in which they featured, particularly in the areas of social and emotional support for individuals living with chronic illnesses.

On a related note, the extensive experience with telehealth provides many lessons for researchers seeking to develop, or health care providers seeking to use, technology to reduce the distance between providers and users. Privacy concerns featured in most studies, with communities citing serious concerns about the social and legal impacts of potential confidentiality breaches. A further issue communities faced was lack of infrastructure; in some cases, the technology that had been developed could not be implemented due to insufficient broadband networks. Finally, major recommendations for continued use of telehealth and similar technologies emerged. First, that health care providers and users receive adequate training on the operation of the system, and second, that uses for the system be diversified beyond health to ensure maximum uptake. With a similarly small sample, the 5 studies concerning assistive devices presented some very real, practical concerns facing Indigenous users of assistive technology. Users expressed several access issues: remoteness impacted maintenance of the device, cost was prohibitive in many cases, and there was often stigma around using the devices. Mentioned as a key concern in 4 of 5 studies was the failing of devices to be sufficiently durable for the frequent and prolonged outdoor activity that remains essential to the lives of many older Indigenous adults.

### Limitations

Despite a review of the published and gray literature, there remained several gaps. Research on the prevalence, policy context, and perception of assistive technology use, particularly mHealth, by Indigenous peoples in Canada was still in early stages. Notably, Inuit and Métis perceptions were not captured in the search. There was also no literature on assistive technology development to support Indigenous caregivers of older adults. Finally, there was no Indigenous-specific research on assistive technology for people with cognitive impairments such as memory loss, an emerging subset of mainstream assistive technology. In particular, further study should be conducted on culturally rooted perceptions of surveillance technologies used to support older adults with dementia, as this technology has the potential to replicate cycles of oppression and colonization.

There is also a considerable gap in evaluated products [39]. In Australia, researchers partnered with the Metro North Hospital have adapted a cardiac rehabilitation app for use by Aboriginal and Torres Strait Islander patients using similar principles, though they caution that the technology remains new and an evaluation has not yet been conducted [38]. Similarly, Brusse and colleagues (2014) caution that further evaluation is needed before conclusions can be drawn, and Shand et al (2013) have published a protocol to determine effectiveness of suicide intervention apps for Indigenous Australian youth [44].

### Conclusions

Overall, technology use, adaptation, and development by Indigenous users existed prominently in the context of colonial legacy. Users had high health needs related to early onset of age-related conditions, multiple comorbidities, and a long history of inadequate health service delivery. Of the studies included in the review, those that empowered communities to direct technology development processes and those that built on existing strengths were most successful. The results also suggest that technology that is highly adaptable to task, context, or culture appear to show the greatest acceptance by Indigenous communities across the globe. Although minimization of the digital divide is also a critical factor, the ability for adaptation of technology may underlie the clear superiority of mHealth versus telehealth. The mHealth initiatives were all specifically adapted for use with Indigenous communities, many with meaningful user consultation. Telehealth, in contrast, was a platform developed for majority culture users and attempted with Indigenous users. Molyneaux and O’Donnell (2009) underscore the need for telehealth use to be diversified beyond use merely for health care within a community to maximize adoption and increase perceptions of cultural safety [13].

This review underscores the importance of exploration of the needs of unique populations, such as Indigenous communities. Technology development in collaboration with Indigenous communities rather than dissemination in those communities is critical for uptake. The incorporation of community-based participatory research methods as a means to inform technology develop met with the greatest success. mHealth platforms codeveloped with Indigenous communities offer a model for future technology development, and we suggest this is an area for future research. Moreover, mHealth platforms can capitalize on large corporate Mobile phone interests to develop a distributed infrastructure, at least with larger and urban centers. Nevertheless, cell tower networks need to be significantly expanded to encompass Canada’s most vulnerable and remote communities. We suggest government policy needs to address mobile phone inequities in low density populations to further reduce the digital divide for Indigenous users of mHealth.

## Abbreviations

mHealth: mobile health
PAR: participatory action research
SMS: short message service
